# Evolution of small molecule-mediated regulation of arbuscular mycorrhiza symbiosis

**DOI:** 10.1098/rstb.2023.0369

**Published:** 2024-11-18

**Authors:** Pierre-Marc Delaux, Caroline Gutjahr

**Affiliations:** ^1^ LRSV, Université de Toulouse, CNRS, UPS, Toulouse INP, 31326 Castanet-Tolosan, France; ^2^ Max-Planck-Institute of Molecular Plant Physiology, Potsdam Science Park, Am Mühlenberg 1, Potsdam-Golm 14476, Germany

**Keywords:** strigolactone, pigment, chitin oligomer, common symbiosis pathway, root

## Abstract

The arbuscular mycorrhizal (AM) symbiosis formed by most extant land plants with symbiotic fungi evolved 450 Ma. AM promotes plant growth by improving mineral nutrient and water uptake, while the symbiotic fungi obtain carbon in return. A number of plant genes regulating the steps leading to an efficient symbiosis have been identified; however, our understanding of the metabolic processes involved in the symbiosis and how they were wired to symbiosis regulation during plant evolution remains limited. Among them, the exchange of chemical signals, the activation of dedicated biosynthesis pathways and the production of secondary metabolites regulating late stages of the AM symbiosis begin to be well described across several land plant clades. Here, we review our current understanding of these processes and propose future directions to fully grasp the phylogenetic distribution and role played by small molecules during this ancient plant symbiosis.

This article is part of the theme issue ‘The evolution of plant metabolism’.

## 1. Introduction

In extant terrestrial ecosystems, most land plants engage in a mutualistic interaction with fungi from the Glomeromycotina [1], forming the arbuscular mycorrhizal (AM) symbiosis [2]. During AM, the two partners exude symbiotic signals into the soil, leading to the activation of specific genetic programmes. Following this mutual recognition, the fungi penetrate the root of host plants, or the thallus in the case of non-vascular plants, and form intra-cellular structures called arbuscules. These highly branched structures are surrounded by the plant plasma membrane and are the sites of nutrient exchange between the two partners. The host plant provides carbon, mostly lipids and carbohydrates, and the AM fungus provides phosphate, nitrogen and other minerals and water collected from the surrounding soil. In other words, the fungus works as an extension of the plant body, mining the soil and improving plant nutrition [2]. Decades of genetics in model flowering plants, such as *Lotus japonicus*, *Medicago truncatula* or rice, have led to the discovery of genes and proteins regulating the different steps of the AM symbiosis, demonstrating that the plant itself regulates the progression of the fungus inside the root and, together with the fungus, builds the interface [3]. The discovery of plant macrofossils harbouring arbuscule-like structures [4], and the occurrence of AM symbiosis in most extant land plants, both from the vascular and non-vascular plant lineages, supported the hypothesis that AM was already present in the most recent common ancestor of all land plants [5]. Reverse genetic analyses recently demonstrated that AM is regulated by orthologous pathways in flowering plants and in an emerging model liverwort, *Marchantia paleacea*, finally demonstrating the ancestral nature of AM [6,7]. Indeed, the parsimony principle states that any trait controlled by the same genetic pathway in vascular plants and non-vascular plants was likely already present in their most recent common ancestor, a relative of the first land plants [8]. Because of this ancient origin, it is possible to track symbiotic pathways using comparative phylogenomics (i.e. comparing genomes in a phylogenetic context). Such approaches have revealed multiple candidate genes awaiting reverse genetic validation [9,10].

By contrast with the genetic pathways involved in AM across land plants, identifying and characterizing the role played by small molecules remains extremely challenging. In this review, we discuss our current understanding of the role of metabolites in AM symbiosis and the conservation or diversification of these across land plants. Doing this, we focus on those molecules that either appear to play unique roles in AM symbiosis or for which most knowledge is available in an evolutionary context. Finally, we propose directions that may provide a more global picture of the small molecules that have been involved in AM symbiosis since the first land plants emerged on land 450 Ma.

## 2. The common symbiosis signalling pathway

Genetics in flowering plants has identified a common symbiosis signalling pathway (CSSP) essential for the activation of the plant symbiotic programme, both in the context of the nitrogen-fixing root nodule symbiosis and the AM symbiosis [11]. This pathway includes a receptor-like kinase (SYMRK/DMI2, [12,13]), cation channels localized in the nuclear envelope (CASTOR and POLLUX/DMI1 [14,15]), a nuclear-localized calcium and calmodulin-dependent protein kinase (CCaMK/DMI3 [16]) and a transcription factor (CYCLOPS/IPD3 [17,18]). Activation of SYMRK/DMI2 following perception of symbiont-derived signals by the plant cell triggers the activation of calcium oscillations in and around the nucleus, which require CASTOR and POLLUX/DMI1 [19]. These oscillations are thought to be decoded by CCaMK/DMI3, leading to its activation and the phosphorylation of CYCLOPS/IPD3. Once phosphorylated, CYCLOPS/IPD3 binds to the promoter of its target genes, leading to the induction of symbiotic responses [20]. Beyond the legume species in which this pathway was first discovered, reverse genetics in monocots have extended their symbiotic function to the entire flowering plant lineages [17,21,22]. Phylogenetic analyses indicate that bryophytes, which diverged from the vascular plant lineage approximately 450 Ma, possess the CSSP genes [23,24] and that all these genes have been exclusively maintained in plant lineages able to form intra-cellular symbiosis, while plant species that have lost symbiotic abilities have also lost some or all of them [9,10,25]. Furthermore, finally demonstrating the ancestral nature of this pathway in land plants, mutants of the liverwort *M. paleacea* affected in SYMRK/DMI2, CCaMK/DMI3 or CYCLOPS/IPD3 are unable to associate with the AM fungus *Rhizophagus irregularis* [26]. Altogether, these studies demonstrate that the CSSP, which is activated in the presence of AM fungi, has been essential for symbiosis for 450 Myr. Conceptually, activation of the CSSP should require plant receptors upstream of SYRMK/DMI2 and fungal signals activating these receptors.

### (a) Which receptors act upstream of the common symbiosis signalling pathway?

This question has been extensively addressed in the context of another symbiosis, the nitrogen-fixing root nodule symbiosis, which is also dependent on the CSSP. In that context, lysin-motif receptor-like kinases (LysM-RLK) have been demonstrated as essential to perceive bacteria-released symbiotic signals and to activate the CSSP. Indeed, mutants affected either in NFR1/LYK3 or NFR5/NFP do not associate anymore with nitrogen-fixing bacteria, and most of the symbiotic responses are abolished [27–30]. Because the function of the CSSP is conserved between the nitrogen-fixing root nodule symbiosis and the AM symbiosis, these types of receptors are also good candidates for the activation of the CSSP by AM fungi. However, although a number of *lysm rlk* mutants show reduced colonization by AM fungi or quantitative defects in arbuscule formation, none of the single, double or triple mutants affected in members of this gene family have phenocopied the AM symbiosis defect of CSSP mutants until very recently [31–33]. Two hypotheses may explain this discrepancy. Either the gene family is so redundant in angiosperms that higher order mutants are required to detect a CSSP-like AM symbiosis phenotype or another class of receptors is important for the activation of the CSSP. These two hypotheses are not mutually exclusive. Compared to the dozens of LysM-RLK paralogs in angiosperms, the liverwort *M. paleacea* contains only four members in the gene family, reducing the likelihood of functional redundancy [31]. Three of these proteins are LYKs that contain a predicted active kinase, while one is a LYR whose kinase is predicted not to be active. Building on this model, Teyssier *et al*. generated single mutants for each of the four members and quantified their AM symbiosis defect. While three of the mutants were colonized to a level similar to wild-type plants, mutants in the *LYKa* gene resulted in a complete lack of colonization [31], mimicking the CSSP mutants [26]. Higher order mutants in the legumes *L. japonicus* [33] and *M. truncatula* [32], namely the *lys6lys7* and *lyk8cerk1* double mutants, respectively, very recently revealed the essential LysM-RLKs in these model legumes. Altogether, the phenotypes observed in LysM-RLK mutants in flowering plants and the absence of colonization in the *M. paleacea lyka* mutant indicate that the LysM-RLK gene family has played an essential role in AM symbiosis since the most recent common ancestor of the land plants [31–33].

### (b) What are the arbuscular mycorrhizal fungi-derived signals activating the common symbiosis signalling pathway?

By analogy with the symbiotic signals produced by nitrogen-fixing bacteria, the Nod factors [34], it had been hypothesized that AM fungi-derived signals could be lipo-chitooligosaccharides (LCOs). Such molecules have been purified and chemically characterized from diverse AM fungi [35,36]. One hallmark of these molecules is their ability to trigger SYMRK/DMI2 and CASTOR and POLLUX/DMI1-dependent calcium oscillations [37]. Genre *et al.* [37] identified COs, short chitin fragments containing either four or five N-acetylglucosamine residues, as another class of chitin-based signals derived from AM fungi with the ability to induce calcium oscillations. Mining *in vitro* cultures of phylogenetically diverse fungi it was later found that neither LCOs nor COs are specific to AM, or even symbiotic, fungi. Instead, at least one of them was found to be produced by 52 fungal species from five fungal phyla and to act as inducers of fungal developmental responses [36]. Lastly, by monitoring receptor activation following treatment with diverse molecules, Teyssier *et al.* [31] showed that a *M. paleacea* mutant in the *lyr* gene does not respond to either COs or LCOs but forms normal AM symbiosis. This suggests that additional fungal signals may be involved in triggering the symbiotic programme, at least in *M. paleacea*. Further analyses of extracts from AM fungi should reveal the nature of these unknown signals in the future. EPR3, a LysM-RLK able to bind diverse types of non-chitin-based polysaccharides, has been described in the legume *L. japonicus*, and mutants in the paralog *EPR3a* show some AM symbiosis defects [38]. Interestingly, one of such compounds has been detected in the cell wall of AM fungi suggesting that, rather than a soluble compound, the missing signal could be a constituent of the fungal cell wall [38].

## 3. Promotion of arbuscular mycorrhizal symbiosis by carotenoid-derived molecules

### (a) Activation of the fungus by strigolactones was established in the earliest land plants

Colonization of angiosperm roots by AM fungi is aided by plant metabolites exuded into the rhizosphere, which activate the fungus and likely act in symbiont recognition and as a directional signal. The so-called ‘branching-factors’, which induce fan-like branching of fungal germinating hyphae [39] and the production of COs [37], were identified to be strigolactones (SLs) [40,41]. Exuded SLs are required for efficient root colonization of angiosperms such as rice, pea, *M. truncatula*, tomato or *Petunia hybrida* and mutants defective in SL biosynthesis or exudation show strongly reduced root colonization [42–44]. The structure of SLs varies among plant species and plants usually produce a number of different SL structures. It is yet unknown which evolutionary drivers may have led to the diversification of these structures. One possibility is the escape from recognition by seeds of parasitic plants, for which SLs act as a germination stimulant, or SLs may have sub-functionalized to attract different soil microbes (reviewed by Kee *et al*. [45]).

Canonical SLs of the strigol and orobanchol type are characterized by an enol-ether bridge, connecting a variable tricyclic lactone ring (ABC ring) to a single lactone ring (D-ring), whereas non-canonical SLs (e.g. heliolactone, zealactone, lotuslactone and avenaol) lack the A, B or C ring (summarized in Yoneyama & Brewer [46]). A number of naturally occurring and synthetic SL structural variants induce hyphal branching of the AM fungus *Gigaspora margarita*, but interestingly, the branching pattern differs for some compounds [47]. This suggests that the cocktail of SL types exuded by plants may influence the morphology and possibly functioning of AM fungi and that certain cocktails may confer an advantage over others. All four rings were necessary to evoke a strong branching response, but the enol-ether bond between the C and D rings could be replaced by other ethers [47]. *G. margarita* also responded to the non-canonical SLs lotuslactone, avenaol, heliolactone, zealactone and methyl-carlactonoate but less strongly than to canonical SLs such as 5-deoxystrigol [48]. While the SL structural requirements for hyphal branching responses have not been investigated for other AM fungal species, this suggests that the fungal SL receptors may have evolved a certain degree of flexibility towards their ligands. Alternatively, receptor gene duplication may have allowed for sub-specification of affinities towards ligands. In fact, there is circumstantial evidence for different fungal receptor systems triggering hyphal branching. *Eustoma grandiflorum* (Gentianaceae) exudes the monoterpene glucosides gentiopicroside and swertiamarin in response to treatment with the plant hormone gibberellin (GA) and these induce hyphal branching of *R. irregularis* and *R. clarus* belonging to the order Glomerales in a similar manner as the SL analog GR24 [49]. However, they do not induce hyphal branching of *G. margarita*, which is a member of the order Diversisporales [49], suggesting that the Glomerales’ receptor is either more flexible or that Glomerales have evolved at least two separate types of receptors for SLs and monoterpene glucosides. An AM fungal SL or monoterpene glucoside receptor has not been identified. Based on structural homology to the plant α/β-hydrolase receptor DWARF14 (D14), a candidate SL receptor from the plant pathogenic fungus *Cryphonectria parasitica* was proposed [50]. *C. parasitica* responds with decreased colony growth to the synthetic SL GR24^5DS^, so the response is quite different from AM fungi. In deletion mutants of the so-called *CpD14* (although it is not an ortholog of the plant D14 gene), the response is reduced. The CpD14 protein binds and hydrolyses synthetic SL *in vitro*, consistent with a role as an SL receptor [50]. It will be interesting to learn about the nature of AM fungal SL receptors, if the perception of SL in plants and symbiotic fungi evolved convergently in the α/β-hydrolase receptor clade or whether in AM fungi, SL receptors have evolved in a different protein family.

In angiosperms, SLs are synthesized from all-trans-β-carotene, which is converted to carlactone by a carotenoid isomerase called DWARF27 (D27) and two CAROTENOID CLEAVAGE DEOXYGENASES 7 and 8 (CCD7 and CCD8) [51]. A cytochrome P450 CYP711A called MORE AXILLIARY GROWTH1 (MAX1) further modifies carlactone to carlactonoic acid (CLA). Downstream of CLA, the pathway diversifies among plant species and CLA is further modified to a number of SL-types (depending on the plant species) by CYP711A or similar cytochrome P450 enzymes (summarized in Nomura *et al*. [52]). The mechanisms for SL diversification are not fully and comprehensively understood and probably yet unknown enzymes await their discovery.

**Figure 1.**
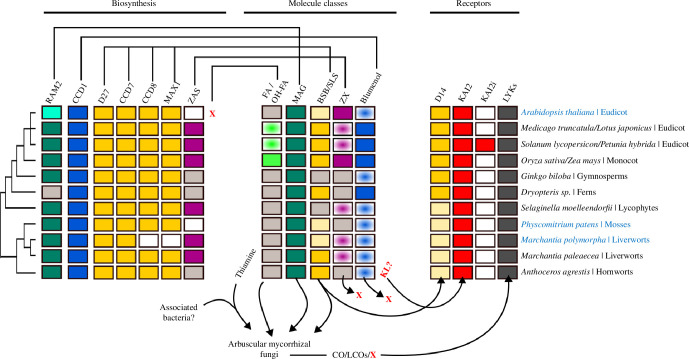
Phylogenetic distribution of AM-relevant metabolites, their biosynthesis and receptor genes. Colours indicate the presence of the indicated compound or gene in the respective plant. White indicates experimentally or computationally determined absence, while grey indicates ‘not tested’. Experimental evidence for bryosymbiol (BSB) or canonical strigolactones (SL) biosynthesis is shown in dark yellow, while the absence of BSB and canonical SL is shown in light yellow. Shaded colours indicate presence of homologs but not orthologs (i.e. turquoise for *Arabidopsis RAM2*). Halos indicate the presumed presence of a given compound based on the presence of biosynthesis genes in the genome. Plant species on the right are examples of their clade, which are subject to prominent research activity. AM-incompetent plants are written in blue. *D27*, *DWARF27*; *CCD7* and *8*, *CAROTENOID CLEAVAGE DIOXYGENASE 7* and *8*; *MAX1*, *MORE AXILLIARY GROWTH 1*; *ZAS*, *ZAXINONE SYNTHASE*; *D14*, *DWARF 14*; *KAI2, KARRIKIN INSENSITIVE 2*; *RAM2*, *REQUIRED FOR ARBUSCULAR MYCORRHIZATION 2*; BSB, bryosymbiol; CO, chitin oligomer; LCO, lipochitoologosaccharide; (OH-)FA, (hydroxy) fatty acid; MAG, monoacyl-glycerol; KL, KAI2-ligand; LYK, LysM-receptor-like kinase; SL, strigolactone; X, unknown compound or gene.

SL biosynthesis genes have not been detected in algal genomes, but *D27*, *CCD7*, *CCD8* and *MAX1* are present in the genomes of bryophytes, ferns, lycophytes and gymnosperms (figure 1; summarized in Nomura *et al*. [52]), indicating that SL biosynthesis may have evolved in the most recent common ancestor of the land plants, thus one of the earliest land plants. A non-canonical SL bryosymbiol (BSB) was discovered in *M. paleacea* [6]. Interestingly, BSB is produced in at least three AM-competent *Marchantia* species, and was also found in exudates of an AM-competent hornwort and two ferns, the monocotyledon asparagus and the dicotyledon peanut. In contrast, it is not produced by *Marchantia polymorpha* and the moss *Physcomitrium patens*, which both lost either *CCD8* and *MAX1* or only *MAX1*, respectively, consistent with their loss of the ability to form AM (figure 1; [6]). Thus, BSB may be an ancient SL type, which has been retained across plant evolution. *M. paleacea ccd8* double mutants were unable to synthesize BSB and most thalli were not at all colonized by AMF, a phenotype reverted with the addition of exogenous SLs. This indicates that SL-like molecules were likely required for AM fungal activation already in the common ancestor of bryophytes and vascular plants, the earliest AM-forming land plants. Angiosperm *ccd8* mutants such as in rice and pea show reduced levels of root colonization [42,43,53] and thus a less severe phenotype than *M. paleacea ccd8* double mutants. This suggests that angiosperm roots may exude additional compounds with activating effects on AM fungi, while *M. paleacea* may majorly rely on BSB.

In angiosperms, SLs were proposed to function as hormones and play an important role in the inhibition of shoot branching [43,54] and other developmental phenomena such as secondary stem growth, root development and senescence (reviewed in Umehara *et al*. [55]). The gene encoding the α/β-hydrolase SL receptor D14 evolved in angiosperms and is absent from bryophytes (figure 1; [56,57]). Consistently *M. paleacea ccd8* double and *max1* mutants do not show developmental defects or other significant changes [6]. This suggests that SL-like molecules such as BSB have initially evolved for exudation and communication with the biotic environment, i.e. to attract AM fungi and possibly other soil microbes, and/or to act in plant–plant communication [6]. A precursor of non-canonical and canonical SLs or a metabolite produced by a branch of the pathway may then have been recruited as a ligand for D14. This notion is supported by three lines of evidence: (i) a tomato mutant in *CYP722C,* which acts downstream of *MAX1*, showed reduced orobanchol and solanachol levels in the exudate with no effect on shoot branching [58]; (ii) genetic and functional characterization of the quadruplicated *MAX1* genes in rice showed that the last step of canonical SL biosynthesis can be abolished without an effect on shoot branching [59]; and (iii) adding an SL receptor to *M. paleacea* is sufficient to synthetically evolve SL perception and responses [6]. Together these indicate that canonical SLs may act exclusively as rhizosphere signals also in angiosperms.

Upon phosphate and nitrogen starvation, SL biosynthesis is enhanced in angiosperms [60–64], suggesting that SL exudation may have evolved to promote AM symbiosis as an adaptation to low nutrient conditions. Consistently, the expression of SL biosynthesis genes is controlled by master regulators of phosphate starvation responses called PHOSPHATE STARVATION RESPONSE belonging to the MYB transcription factor family [63]. In addition, a complex of two GRAS transcription factors NODULATION SIGNALLING PATHWAY 1 and 2, which are themselves transcriptionally regulated by PHRs, regulates SL biosynthesis genes at low phosphate conditions [63,65]. *NSP1* and *2* have originally been discovered to be required for the development of root nodule symbiosis of *M. trunactula*, as mutations in these genes completely abolish nodulation [66,67]. In the same species they were later found to be also necessary for expression of SL biosynthesis genes [68]. The wiring of SL biosynthesis to the phosphate starvation response system to activate AM fungi upon phosphate shortage seems to have occurred early in evolution, as in *M. paleacea* and the hornwort *Anthoceros agrestis*, SL biosynthesis genes are induced in limiting phosphate conditions [6]. *PHR* as well as *NSP1* and *2* genes are present in the *M. paleacea* genome and it will be interesting to understand whether they directly regulate SL biosynthesis genes in these lineages that diverged from the vascular plants more than 400 Ma.

### (b) Additional apocarotenoids and unknown molecules promote intraradical colonization

Another type of apocarotenoid called zaxinone has been discovered in rice and was also detected in tobacco and *Arabidopsis* [69]. It is synthesized by a CAROTENOID CLEAVAGE DEOXIGENASE, which was termed zaxinone synthase (ZAS). The rice genome contains four ZAS paralogs (called ZAS, ZAS1b, ZAS1c and ZAS2). *ZAS* and *ZAS2* genes are induced upon phosphate starvation and single mutants of *ZAS* and *ZAS2* display reduced levels of root colonization suggesting that a ZAS product promotes colonization [69–71].

The role of zaxinone has not been described in AM-competent plants other than rice. However, *ZAS* is generally present in genomes of AM-competent plants including the liverwort *M. paleacea* (figure 1; [25,69]), but it is absent from the non-host *Arabidopsis* genome. Nevertheless, *Arabidopsis* is capable of synthesizing zaxinone via an unknown and (potentially partially non-enzymatic) pathway [69,72]. In *Arabidopsis* roots, zaxinone promotes strigolactone and abscisic acid biosynthesis [70], while in rice, it seems to suppress strigolactone biosynthesis [69]. Thus, it appears that the wiring of the yet unknown zaxinone receptor or the role of zaxinone may have diversified among plants or zaxinone may act differently depending on the plant’s physiological state.

*KARRIKIN INSENSITIVE 2* is the ancestral paralog of the strigolactone receptor gene *D14* that evolved before the diversification of the streptophytes (figure 1; [56,57]). It acts as a receptor of a metabolite synthesized from karrikin, a butenolide compound formed in the smoke of burning vegetation, which induces the germination of fire-following plants [73]. There is ample circumstantial evidence that KAI2 must bind (an) endogenous compound/s, which remain/s to be identified (reviewed in Varshney & Gutjahr [74]).

It is likely that the KAI2-ligands comprise a family of diversified compounds (or even entirely divergent compounds) as diversification of the binding pocket has been observed in KAI2 isoforms of *P. patens*, *L. japonicus*, *Brassica tournefortii* and representative plants of the lamiid subclade [75–79]. This hypothesis has now been supported by the discovery that the volatile sesquiterpene (-)-germacrene D is perceived by the lamiid-specific KAI2ia in *Petunia* to regulate stigma development and reproduction, while the representative of the widely conserved KAI2 clade KAI2ca was unable to perceive this compound [77].

*KAI2* is required for root colonization by AM fungi in rice, *Petunia hybrida*, *M. truncatula*, *Brachypodium distachyon* and barley [21,80–82]. Mutating one of the conserved *KAI2* paralogs (here called *KAI2a*) in *Petunia* was sufficient to abolish AM symbiosis [82], making it unlikely that (-)-germacrene D acts as a KAI2-ligand regulating AM. Interestingly, KAI2 does not play a role in AM in the liverwort *M. paleacea,* suggesting that it was wired to the regulation of AM or phosphate starvation signalling, of which AM-signalling is part, during the evolution of the vascular plant lineage [6,63,83]. When this connection was established during plant evolution is unknown. Nevertheless, evidence from transcriptomes of *smax1* mutants, which are defective in the target of KAI2 and the SCF^MAX2^ complex SUPPRESSOR OF MAX2 1 (SMAX1), suggests that KAI2 and its unknown ligand prime roots for AM symbiosis by leading to increased expression of strigolactone biosynthesis genes and CSSP genes required for fungal entry into the root [84,85]. Activation of the KAI2-pathway by treatment with a KAI2-ligand caused an increase in strigolactone exudation in the AM-hosts rice, maize tomato and *L. japonicus*, but no increase in the typical methyl-carlactonoate in *Arabidopsis thaliana*, which secondarily lost the competence to form AM [86]. This shows that the wiring of KAI2-signalling to AM-relevant genes can also be lost again, although some of the target genes, in this case SL biosynthesis genes, are still present. Interestingly, the activation of KAI2-signalling also did not induce BSB biosynthesis genes in *M. paleacea*, confirming that KAI2 plays no role in promoting AM symbiosis in the liverwort [6,86].

## 4. Promotion of arbuscular mycorrhizal symbiosis by lipids

### (a) Arbuscular mycorrhizal fungi of different orders diversified in their receptor or response systems for exuded fatty acids

Strigolactones do not seem to be the only signals influencing the development of AM fungi outside the root. Plants exude a diversity of metabolites for which an effect on AM fungi is yet unknown. The root exudate includes fatty acids (FAs) and, for example, linolenic acid, lauric acid, oleic acid and 6-hydroxihexanoic acid were found in root exudates of tobacco plants [87]; palmitic acid and stearic acid in root exudates of barley; and adipic acid, palmitoleic acid and oleic acid in root exudates of maize [88].

Several hydroxy-FAs exuded from carrot roots were tested for their activity on hyphal growth of the AM fungus *G. gigantea* [89]. While 3-hydroxydecanoic acid inhibited hyphal growth, 2-hydroxytetradecanoic acid and 2-hydroxydodecanoic acid induced small hyphal branches and stimulated overall hyphal growth, indicating that the structure of hydroxy FAs matters for perception by *G. gigantea*, which belongs to the order Diversisporales (discussed in Kameoka & Gutjahr [90]). Interestingly, *R. irregularis* (formerly *Glomus intraradices*) belonging to the order Glomerales did not respond to 2-hydroxytetradecanoic acid, although its growth was inhibited by 3-hydroxydecanoic acid [89]. This is opposite to the finding that the branched-chain FA (S)−12-methyltetradodecanic acid isolated from the soil bacterium *Paenibacillus validus* as well as the straight-chain FAs C16:1Δ11cis FA, C16:1Δ9cis FA and myristic acid (C14:0 FA) induce hyphal branching and even spore formation in absence of a plant host in *R. irregularis* and *Rhizosphagus clarus* but not in *G. margerita* [91–93]. Together with the finding that the monoterpene glucosides gentiopicroside and swertiamarin induce hyphal branching only in members of the Glomerales but not a representative of the Diversisporales [49], this indicates that different AM fungi clades evolved divergent sets of receptors and/or response systems to various compounds in root (and bacterial) exudates. Given the diversity of chemical pathways and resulting exudate cocktails this may yield a certain degree of specificity or variation in efficiency among the diverse interactions of plant and AM fungal genotypes.

### (b) Biosynthesis and transfer of lipids at the heart of the arbuscular mycorrhizal symbiosis

It is textbook knowledge that carbon is transferred from host plants to AM fungi [2]. Although sugars are transferred to the symbionts [94], the discovery that mutants in AM-specific biosynthesis genes lead to defects in root colonization and AM fungi have lost the fatty-acid synthase complex concomitantly with the gain of their symbiotic lifestyle prompted the community to look for transfer of lipids [94–97]. Using combinations of lipidomics, metabolic engineering and genetics, four studies collectively demonstrated that FAs are specifically synthesized by host plants in cells harbouring arbuscules and transferred to the colonizing AM fungus [98–101]. Biosynthesis of the symbiotic lipids requires several enzymes encoded by genes such as *FatM* or *RAM2* (see review by Kameoka & Gutjahr [90]) whose transcriptional induction in arbuscules-containing cells requires transcription factors from the CBX1 and WRI5 clades [102,103]. Interestingly, the genes encoding the lipid provisioning machinery, and their regulators, have been duplicated in angiosperms, leading to paralogs dedicated to symbiotic functions. These correspond to single pro-orthologs (one gene in one clade is orthologous to multiple paralogs in another clade, here angiosperms) present in bryophytes. The symbiotic transfer of lipids and the role played by the CBX/WRI5 pro-ortholog in that process was extended to the liverwort *M. paleacea* [7]. Because of the lack of duplication for these genes, a dual function as symbiotic and housekeeping genes can be anticipated in bryophytes, as exemplified by the retention of both the regulators and the biosynthetic machinery in *M. polymorpha.* The occurrence of the same mechanisms for the symbiotic biosynthesis of lipids in both vascular and non-vascular plants indicates that their most recent common ancestor, the first land plants, already had this ability. The biosynthesis of lipids is a commonality in the green lineage. By contrasting the lipid metabolism and regulation in green algae and in land plants it will be possible to understand how this intracellular machinery was recruited to specifically export lipids to symbiotic fungi. Kinases have also been recently identified as key regulators of the activation of the lipid regulatory and biosynthesis machinery, such as CKL1 and CKL2 in *M. truncatula* and their pro-ortholog in *B. distachyon* [104] or ARK1 in rice and *M. truncatula* [105]. Both genes have close homologs across the land plants, indicating that the associated regulatory mechanisms might be conserved too.

Beyond the biosynthesis of lipids in arbuscule-containing cells, and its regulation, how these lipids are transferred to the AM fungus remains an open question. Based on genetics, RAM2 seems to be the last enzyme in the biosynthetic pathway, producing *sn2* mono-acyl-glycerols (MAGs, glycerols with a fatty acid on the second carbon).

The two half-ABCG transporters STR and STR2, whose expression during AM symbiosis is conserved in angiosperms and *M. paleacea*, and which localize at the peri-arbuscular membrane [7,25,42,106], as well as other ABCG transporters [101], are putative candidates for transferring MAGs, or a downstream product, to the fungal symbiont [90]. The transport of lipids across the STR/STR2 complex has not been demonstrated to date, probably for technical reasons, given that lipids are not soluble and difficult to use in common transport test systems such as *Xenopus* oocytes or yeast.

## 5. Are there metabolic cues inducing arbuscule development?

Lipid transfer to the fungus is important for arbuscule branching but not for arbuscule initiation [98–101]. In a number of vascular plants including the legume models *M. truncatula* and *L. japonicus* but also corn, wheat, carrot, clover, mung bean and bean, the first arbuscules upon initiation of the symbiosis form in inner cortex cells [107,108]. AM fungi receive not only lipids but also carbohydrates in the form of hexoses from the plant (reviewed in Roth & Paszkowski [109]). It has been suggested that the reason for preferred arbuscule formation in the cortex layer adjacent to the endodermis may be better access to carbohydrates being transported from shoot to root via the phloem in the form of sucrose [107]. Direct evidence for this is yet missing but carbohydrates seem to be used during arbuscule development as starch disappears from arbuscule-containing cells [110]; transcripts of soluble acid invertase and sucrose synthase genes encoding the enzymes cleaving sucrose into glucose and fructose are induced in arbuscule-containing cells [107,111,112]; and knock-down of the AM-induced *M. truncatula Sucrose Synthase 1* gene leads to a reduced formation of arbuscules and increased arbuscule collapse [113].

GRAS proteins of the DELLA family are required for arbuscule formation [114]. DELLAs are targeted by the GA receptor GIBBERELLIN INSENSITIVE DWARF 1 (GID1) together with the SCF^SLY^ complex for ubiquitylation and subsequent proteolytic degradation [115]. Consequently, GA treatment inhibits arbuscule and AM formation at least in four vascular plant species, *M. truncatula*, *L. japonicus*, pea and rice [114,116–118]. In mycorrhizal roots, DELLAs seem to be expressed in the vascular tissue [114] and it has been proposed that they need to move to the cortex to induce arbuscule formation [119]. Although DELLAs could act from a distance, movement to the cortex would make sense because promoters of DELLA-induced genes are active in arbuscule-containing cells in the cortex [114,117]. DELLA movement from the vasculature into the cortex would explain why first the inner cortex cells become competent for arbuscule formation. Most interestingly, it has been shown in *Arabidopsis* seedlings that DELLA accumulates in the presence of sucrose [120]. It is tempting to speculate that this could also occur in the root vasculature of AM-competent plants during root colonization, with DELLA acting as a quantitative indicator of sucrose availability that can be translated into arbuscule development and thus connects arbuscule development with sucrose availability in the root. Also, in thalli of AM-competent *Marchantia* species, arbuscules form in the midrib parenchyma below the photosynthetic epidermis [25,121]. Further, *Marchantia* genomes encode DELLA [25,122]. However, to our knowledge it is unknown how carbohydrates are delivered to the fungus in *Marchantia* thalli, and at which point in plant evolution DELLA acquired its role in promoting arbuscule development. Thus, an analysis of carbon metabolism and transport in mycorrhizal *Marchantia* as well as the AM phenotype of *Marchantia della* mutants are needed. It will also be interesting to understand how DELLA abundance is regulated in *Marchantia*. It has long been thought that liverworts do not produce GAs, but recently, GA-biosynthesis genes as well as the GA precursors *ent*-kaurenoic acid and GA_12_ were identified in *M. polymorpha* [123]. Although mutations in GA_12_ biosynthesis genes lead to phenotypic alterations in response to far-red light, *Marchantia* species lack canonical GA-receptors [123]. It is thus likely that alternative perception systems mediate responses to GA precursors and it remains unclear whether these involve the degradation of DELLA.

## 6. Pigmented metabolites play a role in regulating an established arbuscular mycorrhizal symbiosis

While the nature and perception of symbiotic signals involved in the earliest and latest steps of the AM symbiosis, such as strigolactones or fatty acids, have been intensely studied, metabolic pathways likely play roles at other steps of the association. A striking feature of root colonization by AM fungi in certain plant species is the accumulation of a yellow pigment, the apocarotenoid mycorradicin, visible enough to be used for phenotyping in large-scale forward genetic screens [124,125]. Another class of apocarotenoids, blumenols, has also been found to accumulate in the roots, but also in the shoots, of diverse plant species [126]. Using RNAi on *Carotenoid Cleavage Dioxygenase 1* (CCD1) in *Nicotiana attenuata* You *et al.* [127] managed to strongly reduce the production of blumenol during the association with AM fungi. While the total colonization level was not affected in these lines, the functionality of the arbuscules seemed compromised, as exemplified by the limited induction of symbiotic marker genes, such as the phosphate transporter *PT4* or the ammonium transporter *AMT*, and a reduced number of vesicles, the lipid-storing structures formed by AM fungi [127]. Beyond angiosperms, blumenol A has been detected in the rhizome of the fern *Dryopteris crassirhizoma* [128], which belongs to a genus generally considered a host for AM fungi [129]. Although the presence of blumenol C, and the link between blumenol C and AM symbiosis remains to be explored outside of the angiosperms, its role as a general regulator of arbuscule functioning represents an exciting hypothesis.

In the liverwort *M. paleacea*, the *CCD1* ortholog is not positively regulated during AM symbiosis [7]. Although it cannot be excluded, the biosynthesis of blumenol during colonization of *Marchantia* is not expected. Intriguingly, a purple pigment accumulates specifically in the area of the *M. paleacea* thallus where arbuscules are formed [7,130]. Induction of the pigment is independent of the presence of arbuscules, as exemplified by the *M. paleacea wri* mutant which is colonized but does not host arbuscules and accumulates the pigment [7]. The nature of the pigment(s) and, more importantly, its function await structural and functional characterization. Interestingly, a purple pigment, the auronidin derivative riccionidin A, also accumulates in the non-host liverwort *M. polymorpha* upon nutrient deprivation [131]. Auronidin derives from the flavonoid pathway, via the chalcone isomerase-like enzyme and specific polyphenol oxydases [132]. Homologs of these enzymes are up-regulated during AM symbiosis in *M. paleacea* [7]. One may hypothesize that the purple pigment observed in the colonized area of the *M. paleacea* thallus is an auronidin-related compound that accumulates in response to a local phosphate-starvation response. It has been demonstrated in angiosperms that the master regulator of the phosphate starvation response, PHR, is essential to activate symbiotic pathways, and pathways known to be activated in arbuscule-containing cells [63,133]. Although functional analyses are essential to test this hypothesis, the putative function of metabolites of diverse nature in vascular plants (blumenols) and liverworts (purple pigment) during arbuscule maturity appears to be in sharp contrast with the metabolites acting at early stages (strigolactones) or during the trophic exchanges (lipids). This might reflect differences between ancient mechanisms that were essential for the AM symbiosis to evolve, and more recently evolved ones, selected as fine-tuner of the symbiotic system.

## 7. Which metabolites acting in arbuscular mycorrhizal symbiosis are missing?

The involvement of metabolic cues, either ancient and conserved or lineage-specific, acting at different steps of AM symbiosis establishment and functioning has been clearly demonstrated. One can anticipate that many more metabolites contribute to the functioning and maintenance of the AM symbiosis. Mining AM fungal genomes revealed that thiamine (vitamin B1) biosynthesis was lost in the most recent common ancestor of the AM fungi [96,97,134]. The recent production of asymbiotic spores including the addition of thiamine in the culture media demonstrated that thiamine can be taken up by AM fungi from the external medium [91,93]. How AM fungi obtain their thiamine during symbiosis is so far unknown. Similar to the loss of the fatty acid synthase which has been compensated by the evolution of a lipid-provisioning pathway on the host plant side, the host could directly transfer thiamine. Alternatively, thiamine could be obtained from associated microorganisms such as bacteria. Endobacteria associated with some AM fungi also lack the thiamine biosynthesis pathway ruling out their potential involvement in this process [135]. By contrast, the rich diversity of hyphosphere-associated bacteria represents a possible source of thiamine [136]. In a different context, the ascomycete *Aspergillus nidulans* has been found to obtain thiamine from associated *Bacillus subtilis* [137]. A similar bacteria–fungus interaction could be envisaged for AM fungi.

An interesting additional layer of complexity was brought up by the cereal *nope1* mutant which shows a strongly reduced association with AM fungi [138]. *NOPE1* encodes for an N-acetylglucoseamine transporter which is conserved across land plants, including bryophytes. The transcriptomic response of AM fungi to exudates from the *nope1* mutant differs significantly from the response to wild-type root exudates, indicating that a compound with an active function is missing from the mutant extracts. The nature of this compound and its actual function remain to be determined.

Beyond the symbiotic metabolites expected from our current knowledge, untargeted comparative metabolomics conducted in several angiosperm species in presence of AM fungi revealed dozens of metabolites accumulating in colonized roots [139–141] and the combination of induced metabolites may differ depending on the colonizing fungal species [142]. Functions can be proposed for some of these metabolites. For instance, propionyl- and butyryl-carnitine, which massively accumulate during AM symbiosis in *M. truncatula* [139], could be involved in the export of fatty acids from plastids facilitating the biosynthesis of MAGs to be transferred to the AM fungus. However, most of them remain to be characterized at the structural and functional level. Also, the root exudate can be expected to contain a number of yet uncharacterized metabolites, which may affect the fungus in the rhizosphere either positively or negatively. It was described in a maize diversity panel that some lines strongly promoted growth of the extraradical mycelium, whereas others did not [143]. While this could potentially be ascribed to differences in the efficiency of lipid transfer to the fungus, it is equally possible that variation in the cocktail of root exudate metabolites among maize lines explains the differential promotion of extraradical fungal development. Also, the shoot metabolome changes in response to AM and in a species-specific manner [144]. The emerging combination of reverse genetics, high-throughput metabolomics and NMR-based structure elucidation has the potential to assign a (structure and) function to these metabolites. Such approaches could be conducted in diverse plant species, in order to identify lineage-specific responses and additional core pathways.

On the fungal side, there is accumulating circumstantial evidence that different fungal clades may have evolved different sets of receptors for plant metabolites with putative signalling functions [49,89,92]. Although, to date, AM fungi cannot be genetically manipulated, it may be possible and exciting to identify potential receptors of characterized plant metabolites that act on AM fungi using heterologous systems or *in vitro* approaches.

## Data Availability

This article has no additional data.
